# Perceptions of users and providers on barriers to utilizing skilled birth care in mid- and far-western Nepal: a qualitative study

**DOI:** 10.3402/gha.v7.24580

**Published:** 2014-08-11

**Authors:** Sharad Onta, Bishnu Choulagai, Binjwala Shrestha, Narayan Subedi, Gajananda P. Bhandari, Alexandra Krettek

**Affiliations:** 1 Department of Community Medicine and Public Health, Institute of Medicine, Tribhuvan University, Kathmandu, Nepal; 2 Department of Internal Medicine and Clinical Nutrition, Institute of Medicine, Sahlgrenska Academy, University of Gothenburg, Gothenburg, Sweden; 3 Nepal Public Health Foundation, Kathmandu, Nepal; 4 Nordic School of Public Health NHV, Gothenburg, Sweden

**Keywords:** skilled birth care, utilization, skilled birth attendants, barrier, qualitative, Nepal

## Abstract

**Background:**

Although skilled birth care contributes significantly to the prevention of maternal and newborn morbidity and mortality, utilization of such care is poor in mid- and far-western Nepal. This study explored the perceptions of service users and providers regarding barriers to skilled birth care.

**Design:**

We conducted 24 focus group discussions, 12 each with service users and service providers from different health institutions in mid- and far-western Nepal. All discussions examined the perceptions and experiences of service users and providers regarding barriers to skilled birth care and explored possible solutions to overcoming such barriers.

**Results:**

Our results determined that major barriers to skilled birth care include inadequate knowledge of the importance of services offered by skilled birth attendants (SBAs), distance to health facilities, unavailability of transport services, and poor availability of SBAs. Other barriers included poor infrastructure, meager services, inadequate information about services/facilities, cultural practices and beliefs, and low prioritization of birth care. Moreover, the tradition of isolating women during and after childbirth decreased the likelihood that women would utilize delivery care services at health facilities.

**Conclusions:**

Service users and providers perceived inadequate availability and accessibility of skilled birth care in remote areas of Nepal, and overall utilization of these services was poor. Therefore, training and recruiting locally available health workers, helping community groups establish transport mechanisms, upgrading physical facilities and services at health institutions, and increasing community awareness of the importance of skilled birth care will help bridge these gaps.

Skilled birth care is a key indicator of maternal and neonatal health (1). Globally, the number of estimated maternal deaths decreased between 1990 and 2010, from 543,000 to 287,000, respectively, but the rate of decline is just over half of that required by Millennium Development Goal (MDG) 5 (2). Skilled birth attendants (SBAs) play an important role in reducing maternal and neonatal mortality because they provide timely obstetric and newborn care for life-threatening complications (3). High maternal mortality in Nepal (currently, 281 deaths per 100,000 live births) (4) is largely due to the high prevalence of homebirths and low utilization of SBAs (5) and results mainly from post-partum hemorrhage (32%), hypertensive disorders of pregnancy (25%), and abortion (13%) (6). As a signatory of Millennium Declaration 2000, Nepal is committed to improving skilled birth care and reducing maternal mortality. Nepal aims to achieve MDG 5 by 2015 (1).

The Safe Motherhood Policy (7) aimed to reduce maternal morbidity and mortality resulting from pregnancy and related causes. Today, this amended policy helps develop safe motherhood programs, protocols, and human resources production, focusing mainly on expanding and improving maternal care at central and peripheral health institutions.

In 2005, the Government of Nepal launched the Maternity Incentives Scheme, currently known as the Safe Delivery Incentive Program, to encourage women to use health facility services for childbirth (8). Women who deliver a baby in a health facility receive a pro-rated cash incentive for transportation, based on ecological region. Thus, women who use delivery services at a health institution in the mountain, hill, and terai (plains) regions receive Nepalese rupees (NRP) 1,500, 1,000, and 500 (1 US$ ≈NRP 95 in June 2014), respectively. Importantly, this program provides no-cost delivery services at health facilities in districts ranked low on the Human Development Index.

In 2006, the Government introduced the National Policy on SBAs, which generally aimed to reduce maternal/neonatal morbidity and mortality by ensuring availability, access, and utilization of skilled care at every birth (9). This policy embodies the Government's commitment to train and deploy doctors, nurses, and auxiliary nurse–midwives across Nepal. Also in 2006, the Government launched the National Safe Motherhood and Newborn Health Long Term Plan, which is a revised version of the National Safe Motherhood Long Term Plan. The revised plan aims to increase healthy practices and utilization of quality maternal and neonatal health services among poor and excluded women (10).

In 2009, the National Free Delivery Policy made all institutional deliveries free of charge (11). Successful implementation of this program improved the proportion of institutional deliveries, increased staff attendance at births, and resulted in more active health facility management committees (12).

These health care policies helped reduce maternal mortality from 539 maternal deaths per 100,000 live births in 1996 (13) to 281 in 2006 (4), mainly due to increased utilization of SBAs at delivery [from 10.1% in 1996 (13) to 18.7% in 2006 (4)]. By 2011, the utilization of SBAs at delivery increased to 36% (14). Despite a nationwide increase in SBA utilization, percentages vary across Nepal's five administrative regions. In the mid- and far-western regions, use of SBA services is 28.7% and 30.7%, respectively, which is lower than utilization in the eastern, central, and western regions (14). Such evidence demonstrates the need to identify barriers, understand the perspectives of service users and providers regarding those barriers, and develop strategies to improve women's access to and utilization of delivery care services.

Our recent quantitative study in mid- and far-western Nepal shows that women's knowledge of pregnancy danger signs, household economic status, distance to health facilities, and completed antenatal care visits influence women's decision to use SBAs (15). Therefore, the present study aimed to explore how service users (mothers) and providers (health workers and members of health facility management committee) perceive barriers to skilled birth care and what solutions they perceive to overcome those barriers in mid- and far-western Nepal.

## Methods

### Sampling strategy and study sites

We purposely selected the same districts we used for our quantitative study in mid- and far-western Nepal (i.e. Dailekh, Bajhang, and Kanchanpur) (15). These districts have lower utilization of SBA services (14). Dailekh is located in the hills of mid-western Nepal, whereas Bajhang is a mountain district, and Kanchanpur is a terai (plains) district in far-western Nepal. Each district has different ethnic groups: aboriginal (*Tharu*) people in Kanchanpur, upper caste but economically deprived *Chhetri* and *Bahun* in Dailekh, and *Mongoloid* people in Bajhang. All three districts lag behind the national average regarding educational health and economic development standards. The mountain and hill districts (Bajhang and Dailekh, respectively) have difficult geography and poor road conditions. Compared to Bajhang and Dailekh, the geography of Kanchanpur, a terai district, is easier but its remote areas have less access to transport. Although health facilities extend to the level of Village Development Committee (VDC) in all three districts, access to services is limited due to difficult terrain, poor road conditions, and inadequate transport.

The VDC and municipality are the basic politico-administrative units within these districts. The study districts comprise a mostly rural population and include 55 VDCs and one municipality in Dailekh, 48 VDCs in Bajhang, and 19 VDCs and one municipality in Kanchanpur.

We selected specific communities for focus group discussions (FGDs) based on distance from the district hospital and conducted eight FGDs (four each for service users and service providers) in each district to cover the communities at varying distances from the hospital. Every service-user FGD included at least two mothers who had delivered within the past 12 months and at least one female community health volunteer (FCHV). All other participants were members of mothers’ groups. Each service-provider FGD included health workers, facility managers, and local leaders. Finally, each FGD contained 7–12 participants.

### Study population

Study participants comprised service users and service providers. Specifically, service users included mothers who had delivered a baby within past 12 months and other married women of reproductive age; all were selected from the mothers’ group in each community. Service providers included health workers and members of the Health Facility Management Committee, which included health facility managers and local leaders. Health workers had at least 15 months of formal training in nursing or general medicine and were registered in their respective professional councils.

### Data collection

We recruited and trained eight field researchers, each having a bachelor's degree in public health, to conduct FGDs in the study districts. We also recruited a local resource person to guide the field researchers and inform them about appropriate locations for data collection. To explore the social context, cultural issues, and concerns related to SBA utilization, we conducted 24 separate FGDs during May–June 2011 (12 FGDs with service users and 12 with service providers). We also explored suggestions to improve SBA service utilization.

FGD guidelines included information on users’ and providers’ perspectives on barriers to accessing SBA services, transport issues, and service-side issues (e.g. availability, capacity, and motivation of human resources, health infrastructure, and logistic supply, and the role of the health facility management committee). Some probing questions explored the quality of care and perceived strategies to overcome the barriers.

To establish trustworthiness, we adopted approaches discussed by Krefting (16). We visited and observed the actual physical environment to understand its social and cultural context. We developed separate guidelines for FGDs with health service providers and users, and all field researchers attended a two-day training session. We allowed field researchers the flexibility to probe and explore issues according to the context of the discussion and the participants’ concerns regarding perceived barriers to skilled birth care.

### Data analysis

First, we compiled notes from all 24 FGDs and labeled them according to participant type. After reviewing individual FGD transcriptions, we organized the data and entered them into a computer in the original Nepali language. Second, we thoroughly reviewed the Nepali transcriptions, translated them into English language, and reviewed each translation to understand the meaning of its content. Importantly, the researchers’ experience with conducting FGD research in Nepal helped ensure correct translations (17). Third, during content analysis (18) we used a deductive approach to identify barriers for accessing care according to the Three Delays model (19), which includes delay in (i) seeking, (ii) reaching, and (iii) receiving care. We also added a fourth theme, based on the supply- and demand-side model, to describe perceived strategies to overcome barriers (20) (Table 1). Fourth, we coded the contents of all FGD notes according to the themes of analysis. Finally, we organized all data according to theme and summarized them according to the pattern of findings for all FGDs (21).

**Table 1:** Themes and categories of data analysis

Themes	Categories
1. Delay in seeking care (demand-side barriers: community awareness, and cultural and financial issues)	Level of awareness Tradition, culture, and women's role Financial issues
2. Delay in reaching health facility: Non-health infrastructure issues related to road, transport, and community organization	Geography, road conditions, and transport Family and community support
3. Delay in receiving care (human resources, health infrastructure, and logistic supply)	Human resource availability, capacity, and motivation Health infrastructure and logistic supply
4. Perceived strategy to overcome barriers	Demand-side interventions (health promotion and education programs to improve awareness and reduce cultural barrier) Improving physical access (road access and means of transportation to increase accessibility to health facility) Improving availability of service providers Improving health infrastructure and supply logistics Policy and program interventions for motivation of SBAs

### Ethical considerations

Before conducting each FGD, we explained the nature of the study, its rationale, and the extent of involvement expected from the participants. All respondents signed a written focus group consent form before participating in the FGD. A witness read the informed consent form to illiterate individuals, and those who consented to participate placed their thumbprint on the form, which was signed by the witness. The Nepal Health Research Council and the World Health Organization (Geneva, Switzerland) approved this study.

## Results

### Delay in seeking care (demand-side barriers – community awareness, and cultural and financial issues)

#### Level of awareness

Although skilled birth care was available in peripheral health facilities, many women in our study were unaware of the importance of delivery by SBAs. Indeed, participants did not think it was necessary to go to a health facility for normal delivery until and unless they experienced a serious problem. A service user from Bajhang said: ‘If the labor pain is very serious then we mostly visit the health facility for delivery or we call a skilled birth attendant to come to our home’. Additionally, women and their families could not prepare for an institutional delivery because they were unable to accurately predict the expected date of delivery.

#### Tradition, culture, and women's role

Many women preferred home delivery because they were ashamed to deliver at the health facility, even when it was near their residence. Further, the cultural practice of untouchability (i.e. isolating women during delivery and a few days after childbirth) prevented some women from visiting the health facility and using an SBA. One service user from Bajhang stated: ‘In our culture, recently delivered women are considered impure and thus cannot pass if there is a temple on the way to the health facility’. Some families did not allow women to deliver at the health facility because they feared an evil spirit might haunt the mother and the baby.

Because women were busy with household chores and child care even on the last day of pregnancy, they did not have time to travel to hospital for delivery. Moreover, husbands in the study area seasonally migrated to India in search of work. Thus, many women lacked adequate family support to plan and go to the health facility for delivery.

Women's lack of autonomy in decision making also affected SBA utilization because their husband or head of household decided whether they should access health care. Indeed, some families prevented women from going to the health facility. A health worker from Dailekh said:

Many husbands do not allow their wives to visit the health facility for seeking delivery services because of traditional beliefs although they have money and can manage transportation. Women cannot ignore their husband's decision and cannot express their opinion in the family.

#### Financial issues

Poverty also prevented some women from going to a health facility for delivery. Although women in the mountain district receive NRP 1,500 for each delivery at a health facility, additional fees for transport, food, and living arrangements for an accompanying member can increase the total cost to more than NRP 4,000. If families cannot cover these extra costs themselves, women deliver their babies at home.

If the district health office delays payment, mothers do not receive the funds instantly. In our study, the poorest families could not afford to visit a health facility. A service provider from Dailekh noted: ‘We are able to provide the delivery incentive to the mother only 3–4 months after the delivery’.

### Delay in reaching the health facility (non-health infrastructure issues related to road, transport and community organization)

#### Geography, road conditions, and transport

Distance and poor road conditions made it virtually impossible for many of our participants to reach the health facility. In some instances, families called SBAs to their home and paid extra for that service. Inadequate or inappropriate transport made it difficult for women to reach the health facility. In the rural terai areas, the common means of transport is a bull/buffalo cart; in hills and mountain districts, people carried pregnant women to the health facility on stretchers. In our study, women in all districts faced difficulties in reaching the health facility, especially at night or during the rainy season. One service user from Kanchanpur commented: ‘It is particularly difficult if the labour begins at night. There is no transport facility and the way is dark. In such a situation, how can we go to the health facility?’

#### Family and community support

Formal and informal community groups, including mothers’ groups, FCHVs, and youth groups, helped arrange transportation for pregnant women to reach the health facility. However, when youths migrated temporarily to seek work, arranging transport or finding enough people was even more challenging. Moreover, such transport received less priority during the planting and harvesting seasons. Short duration between the onset of labor and delivery aggravated this situation. However, family members were increasingly supportive in recent years. One service user from Kanchanpur said: ‘These days mothers-in-law in my VDC do not restrict their daughters-in-law to seek delivery services from the health facility’.

### Delay in receiving care (service-side barrier – human resources, health infrastructure and logistic supply)

#### Human resource availability, capacity, and motivation

Both service providers and users mentioned that at least one trained health worker was available in the health facilities. A few facilities had an assistant nurse midwife (ANM) available 24 hours/day because the ANMs stayed nearby in rental apartments. However, many peripheral facilities lacked ANMs. A service user from Bajhang commented: ‘Only one ANM is available in the health facility, thus when she is on leave, in training or transferred to another health facility, we cannot get SBA service from the health facility or in our home’. In some health facilities, the management committee hired local ANMs on a contract basis. Women appreciated locally recruited ANMs due to regular availability.

The scarcity of health workers in health facilities resulted partly from unfulfilled posts. When the number of deliveries exceeded SBAs’ ability to provide services, staff members became overburdened. The absence of a clear division of roles among staff members who shared responsibility for providing maternal health services further aggravated this situation and affected worker motivation and performance. A service provider from Dailekh remarked: ‘We are so busy we cannot manage time for our family even after regular office hours. We do not get adequate time for food and rest. Within the available staff, the workload is not properly divided’.

Because SBAs in peripheral areas were mostly ANMs with limited training, they were unable to handle complicated deliveries. One service provider from Bajhang noted: ‘SBAs are facing problems in cases of difficult labor, breech delivery and occasionally the women have to deliver on their way to the health facility which is very challenging’.

#### Health infrastructure and logistic supply

Inadequate health infrastructure and logistics also posed serious barriers to the provision of SBA services. Health facility buildings were generally small, and the rooms and waiting area were inadequate for the number of deliveries. Water supply, toilets, and privacy in the labor room were frequently inadequate. In some facilities, damaged roofs leaked during the rainy season. Poor light and ventilation were common, and facilities had no alternate source of electricity during power outages (i.e. load shedding). Similarly, limited availability of necessary furniture (including delivery tables), medicines, equipment, and laboratory services hindered SBA services.

The lack of beds and rooms often resulted in the immediate discharge of women and their babies following delivery. A service provider from Dailekh remarked: ‘We were conducting delivery in the open ground. We sometimes ask the local school for furniture. Recently the district health office has given one delivery table’.

Poorly maintained staff quarters negatively affected the regular availability of health workers. One service provider from Bajhang said: ‘Because of a damaged roof we cannot live in the staff quarters during the rainy season’. Another provider from Bajhang said: ‘Some women have travelled a long distance and it is difficult for them to return immediately after delivery. However, due to lack of beds, we do not have any other option than to discharge them immediately’.

### Perceived strategies of service users and providers to overcome barriers

#### Demand-side interventions (health promotion and education programs to improve awareness and reduce cultural barriers)

Women's lack of awareness regarding the importance of skilled birth care was a main reason for low SBA utilization. Most FGDs consistently suggested the necessity of increasing community awareness to promote safe motherhood practices and SBA-assisted delivery. FGDs emphasized the importance of developing future interventions to strengthen health education and health promotion programs and assist women who had difficulty accessing SBA services. A service provider from Dailekh commented that ‘[we] need to organize health awareness programs in all villages. The traditional practices of home delivery could thus be reduced’.

#### Improving physical access (road access and means of transportation to increase accessibility to the health facility)

Measures that improve physical access include proximity of health facilities, track and trail widening, road construction and maintenance, and timely transport. Service users also recommended additional stretchers, incremental transportation incentives to cover the cost of ambulance or porter services, and timely referrals to a higher-level health facility. Service users from Kanchanpur commented that ‘provision of ambulance services on a regular basis with effective referral system is essential. The public transportation is not very helpful outside the city area’.

#### Improving availability of service providers

Locating staff accommodations near health facilities would facilitate continuous availability of service providers and 24-hour delivery service. Service users believed in the importance of providing accommodations and food for visitors during a mother's stay at the health facility.

#### Improving health infrastructure and supply logistics

Service providers suggested maintenance of a continuous water supply system as well as building and roof repairs at the health facility. Health workers wanted improved security (e.g. fencing) for staff quarters and health facilities at night. They also advocated for separate delivery rooms, adequate furniture, and an alternate source of electricity during load shedding.

Service providers suggested the need for timely supply of adequate equipment (e.g. ultrasound, X-ray, and electrocardiograph machines).

#### Policy and program interventions for motivation of SBAs

The skills of SBAs who work in local health facilities should be upgraded to handle difficult deliveries because poor families cannot afford to visit higher-level health facilities. Increased training of SBAs will solve many delivery problems at the local level. Some service providers in Dailekh and Kanchanpur reflected that ‘[the] SBA should be able to manage difficult cases such as breech delivery, because poor mothers cannot afford to visit the referral hospital’.

Work volume requires the recruitment of additional SBAs and support staff. Service providers from all districts suggested the need for a performance-based policy to reward and recognize SBAs. Educational and training opportunities and incentives for night duty would help enhance SBAs’ motivation. Staff positions sanctioned to local health facilities should be fulfilled, including the availability of a medical doctor in primary health centers.

Service providers emphasized the need to align the training program with the job description. In addition, clear guidelines will help management committees upgrade SBAs based on performance appraisals.

## Discussion

### Delay in seeking care (demand-side barriers – community awareness, and cultural and financial issues)

In our study, women and their families thought that skilled birth care is necessary only when complications occur. Further, because of lack of knowledge on expected date of delivery, many women delivered at home or in transit to a health facility even when the facility was nearby.

Other studies also confirm that women's lack of awareness about the importance of skilled birth care is an important barrier to seeking care. In Tanzania, women do not seek skilled birth care because they do not know that such services are available and do not understand the risk involved in traditional birth practices (22). Similarly, an Indonesian study shows that women use health services during delivery mainly due to childbirth complications (23).

A qualitative study among rural women in central Nepal shows similar results – women go to the health facility only if they experience a problem during labor (24). These findings suggest that women do not understand the importance of skilled care at every birth. Likewise, the traditional thinking that an evil spirit might haunt the woman or child on the way to health facility is a barrier of skilled birth care (25).

In Nepal, women traditionally live with their husband's family after marriage. Women have little or no power in their marital home and depend on their mothers-in-law's perception of pregnancy and delivery-care needs (26). Thus, the mothers-in-law's perception of the benefits of delivery services influences women's decision to utilize those services. Earlier studies from Nepal's neighboring countries also report that women's individual autonomy predicts their utilization of skilled birth care. In India, highly autonomous married adolescents are more likely to utilize maternal health services compared to those with low autonomy (27). Similarly, higher female autonomy in rural Pakistan associates positively with institutional delivery (28). In Bangladesh, women are more likely to deliver at home if an influential person advises them to stay home during delivery (29).

Although the core costs for deliveries are now free in Nepal, women and families still prefer home delivery due to peripheral costs (12). In Bangladesh, women delivering at home are more likely to worry about the costs associated with delivery care (29).

### Delay in reaching the health facility (non-health infrastructure issues related to road, transport and community organization)

In our study area some women delivered en route because of poor road conditions, distant health facilities and inadequate transportation. Hence, women in remote areas feared traveling to a health facility to seek SBA delivery service, going only for prolonged labor or complications. Similarly, in rural Southern Malawi some women delivered in transit while walking to a health facility; others delivered at home (30).

In Nepal's rural Kavre district, inadequate transport is a major reason that 30% of women do not use SBAs (31). Moreover, 22.6% of women do not use SBAs because the health center is too far away (31). In Pakistan, about 38% of women who travelled to the nearest health facility on foot delivered at the facility, compared to 49% who travelled to the nearest facility using some form of motorized transport (28). Separate studies in Bangladesh, Afghanistan, and China confirm that distance to the nearest health facility is a barrier to using delivery care (29, 32, 33).

Indonesia, Uganda, and Kenya, where transport facilities and irregularities among health care providers are the main causes of unavailability and poor utilization of SBA services, have reported a similar scarcity of SBAs (23, 34, 35). In these studies, available SBAs were overburdened by volume.

Our finding that some traditional birth attendants advise women to seek skilled birth care at a health facility is encouraging. However, many women still use traditional birth attendants for normal deliveries. They visit a health facility only when they encounter a problem, possibly due to traditional beliefs and unavailability of SBAs in the community.

Male involvement in decision making increases the likelihood that women will seek skilled birth care. Therefore, increased involvement by males and the community in safe motherhood programs contributes importantly to increased utilization (34). Similarly, relatives and friends influence decisions regarding place of birth. Thus, community clubs that focus on sexual and reproductive health would aid efficient communication of health messages.

### Delay in receiving care (service-side barrier – human resources, health infrastructure and logistic supply)

Worker scarcity, professional capacity, and staff motivation negatively affected the provision of skilled birth care at health facilities. In peripheral areas, most SBAs were ANMs with limited training that renders them unable to handle complicated deliveries.

Although SBAs were available in district hospitals, availability decreased in remote health facilities, possibly because isolation and safety issues made SBAs reluctant to work in such areas. Consequently, rural facilities faced problems in hiring and retaining SBAs. Weak human resource management, irregular logistical supplies, and difficult geography demotivated SBAs even further. Frequently, SBAs who were assigned to remote health facilities worked in the district hospital on deputation for a long time. Thus, positions were occupied on paper but vacant in reality. The underlying reasons for inadequate numbers of SBAs in rural health facilities included recruitment policies that did not reflect the population's need for services. Health facility management committees that recruited contract health workers addressed this barrier to some extent.

In our study area, poor health infrastructure and logistic supply negatively affected the provision of skilled birth care at the health facility. Studies in other low-and lower-middle income countries also report that lack of adequate equipment, human resources, and training opportunities for health workers are major constraints in the provision of SBA services (23, 36, 37).

The health facility management committee links people, the health facility, and local government. Such committees should strengthen facility infrastructure by arranging logistics (e.g. medicine, equipment, furniture) for quality care and managing an emergency fund for transport and referral. Such actions are possible only through the collective effort of the community, local health workers, community-based organizations, women groups, FCHVs, local non-governmental organizations, and local authorities.

### Strategies to overcome barriers

Our results suggest that women in rural and remote areas need knowledge about the government-provided incentive for institutional delivery and also about the opportunity to earn an additional NRP 400 by completing four antenatal care visits. Behavioral interventions, such as birth preparedness and complication readiness, help women and their families appreciate the need for institutional deliveries (38). In Tanzania, skilled birth care is 16.8% higher among women who receive counseling that promotes birth preparedness (39). In Bangladesh, Burkina Faso, and Eritrea, increased awareness and birth planning associates with increased utilization of SBA services (40–42). In contrast, two evaluation studies from Nepal suggest no association between increased birth preparedness and delivery at a health facility (43, 44). However, study design might account for such discrepancies.

In our study area, people still support local traditional birth attendants and have conservative attitudes that must be changed through community awareness and behavior change interventions. Such interventions should target family members, especially husbands and mothers-in-law. Depending on need and situation, communities should transport pregnant women to health facilities for institutional deliveries.

Health facilities need timely disbursement of budget allocations for women's government incentives, whereas health workers need timely disbursement of their salaries from the Ministry of Finance. Such payments would improve SBA service provision and utilization. To promote 24-hour service, SBAs who work at night should receive more pay. Finally, health facility management committees should work with local district development committees and VDCs to generate funds for construction, maintenance, and logistical guidance of health facilities.

### Strengths and limitations of the study

This paper provides a balanced exploration of the perspectives of both service users and providers. It also describes both system-and demand-side barriers to skilled birth care. Therefore, the data presented here comprehensively describe our participants’ perceptions of potential barriers to using skilled birth care.

FGDs might limit the confidentiality and anonymity of participants and their views because all participants interact and listen to each other. We described this possible limitation to our participants before asking for informed consent. Another challenge in maintaining FGD confidentiality in rural Nepal involves finding a space where participants can talk freely and privately without others listening in (45). To address this issue, we reserved a room at a local school or health institution.

## Conclusions

Service users and providers perceived inadequate availability and accessibility of skilled birth care in remote areas of Nepal, and overall utilization of these services is poor. Therefore, training and recruiting locally available health workers, helping community groups establish transport mechanisms, upgrading physical facilities and services at health institutions, and increasing community awareness of the importance of skilled birth care will help bridge these gaps.
